# The waist-to-body mass index ratio as an anthropometric predictor for cardiovascular outcome in subjects with established atherosclerotic cardiovascular disease

**DOI:** 10.1038/s41598-021-04650-5

**Published:** 2022-01-17

**Authors:** Chin-Feng Hsuan, Fang-Ju Lin, Thung-Lip Lee, Kai-Chien Yang, Wei-Kung Tseng, Yen-Wen Wu, Wei-Hsian Yin, Hung-I. Yeh, Jaw-Wen Chen, Chau-Chung Wu, The Taiwanese Secondary Prevention for Patients with AtheRosCLErotic Disease (T-SPARCLE) Registry Investigators, Chau-Chung Wu, Wei-Tien Chang, Yi-Heng Lee, Jaw-Wen Chen, Huey-Herng Sheu, I.-Chang Hsieh, Yih-Sharng Chen, Ming-En Liu, Chen-Huan Chen, Lian-Yu Lin, Hung-I. Yeh, Shih-Hsien Sung, Ping-Yen Liu, I.-Hui Wu, Zhi-Hong Wang, Kuan-Ming Chiu, Yen-Wen Wu, Chi-Tai Kuo, Tzung-Dau Wang, Chung-Lieh Hung, Chih-Hsien Wang, Chun-Chieh Wang, Chih-Yuan Wang, Jiann-Shing Jeng, Tsung-Hsien Lin, Hsien-Li Kao, Pao-Hsien Chu, Fang-Ju Lin, Zhih-Cherng Chen, Kuan-Cheng Chang, Wei-Hsian Yin, Wei-Kung Tseng

**Affiliations:** 1 Division of Cardiology, Department of Internal Medicine, E-Da Hospital, Kaohsiung, Taiwan; 2 Division of Cardiology, Department of Internal Medicine, E-Da Dachang Hospital, Kaohsiung, Taiwan; 3 School of Medicine, College of Medicine, I-Shou University, Kaohsiung, Taiwan; 4 Graduate Institute of Clinical Pharmacy and School of Pharmacy, National Taiwan University, Taipei, Taiwan; 5 Department of Pharmacy, National Taiwan University Hospital, Taipei, Taiwan; 6 School of Medicine for International Students, College of Medicine, I-Shou University, Kaohsiung, Taiwan; 7 Division of Cardiology, Department of Internal Medicine, National Taiwan University Hospital, Hospital. No. 7, Chung-Shan South Road, Taipei City, 10022 Taiwan; 8 Department and Graduate Institute of Pharmacology, National Taiwan University College of Medicine, Taipei, Taiwan; 9 Division of Cardiology, Cardiovascular Medical Center, Far Eastern Memorial Hospital, New Taipei City, Taiwan; 10 Division of Cardiology, Heart Center, Cheng-Hsin General Hospital, Taipei, Taiwan; 11 Mackay Memorial Hospital, Mackay Medical College, Taipei, Taiwan; 12 Department of Medical Research and Education, Taipei Veterans General Hospital, Taipei, Taiwan; 13 Graduate Institute of Medical Education and Bioethics, College of Medicine, National Taiwan University, Taipei, Taiwan; 14 Department of Emergency Medicine, National Taiwan University Hospital, Taipei, Taiwan; 15 Division of Cardiology, Department of Internal Medicine, National Cheng Kung University Hospital, Tainan, Taiwan; 16 Division of Endocrinology and Metabolism, Department of Internal Medicine, Taipei Veterans General Hospital, Taipei, Taiwan; 17 Division of Cardiology, Department of Internal Medicine, Linkou Chang Gung Memorial Hospital, New Taipei City, Taiwan; 18 Division of Cardiovascular Surgery, Department of Surgery, National Taiwan University Hospital, Taipei, Taiwan; 19 Division of Cardiology, Department of Internal Medicine, Hsinchu Mackay Memorial Hospital, Hsinchu, Taiwan; 20 Division of Cardiology, Department of Internal Medicine, Taipei Veterans General Hospital, Taipei, Taiwan; 21 Division of Cardiology, Department of Internal Medicine, Hualien Tzu Chi Hospital, Hualien, Taiwan; 22 Division of Cardiovascular Surgery, Cardiovascular Medical Center, Far Eastern Memorial Hospital, New Taipei City, Taiwan; 23 Division of Endocrinology and Metabolism, Department of Internal Medicine, National Taiwan University Hospital, Taipei, Taiwan; 24 Department of Neurology, National Taiwan University Hospital, Taipei, Taiwan; 25 Division of Cardiology, Department of Internal Medicine, Kaohsiung Medical University Chung-Ho Memorial Hospital, Kaohsiung, Taiwan; 26 Division of Cardiology, Department of Internal Medicine, Chi Mei Memorial Hospital, Tainan, Taiwan; 27 Division of Cardiology, Department of Internal Medicine, China Medical University Hospital, Taichung, Taiwan

**Keywords:** Cardiology, Risk factors

## Abstract

Obesity is an independent risk factor for atherosclerotic cardiovascular disease (ASCVD). However, ‘obesity paradox’ is observed in patients with coronary artery disease while defining obesity by body mass index (BMI). The purpose of this study is to identify a better anthropometric parameter to predict cardiovascular events in patients with ASCVD. The study was conducted using the Taiwanese Secondary Prevention for patients with AtheRosCLErotic disease (T-SPARCLE) Registry. A total of 6,920 adult patients with stable ASCVD, enrolled from January 2010 to November 2014, were included, with a mean age of 65.9 years, 73.9% males, and a mean BMI of 26.3 kg/m^2^ at baseline. These patients were followed up for a median of 2.5 years. The study endpoint was the composite major adverse cardiovascular event (MACE), defined as cardiovascular death, nonfatal myocardial infarction or stroke, or cardiac arrest with resuscitation. Multivariable Cox proportional hazards regression showed a significant positive association between waist-to-BMI ratio and MACE (adjusted hazard ratio 1.69 per cm‧m^2^/kg increase in waist-to-BMI ratio, 95% CI 1.12–2.49, *p* = 0.01) after adjusting for potential risk factors and confounders. Traditional anthropometric parameters, such as BMI, weight, waist and waist-hip ratio, or newer waist-based indices, such as body roundness index and a body shape index, did not show any significant linear associations (*p* = 0.09, 0.30, 0.89, 0.54, 0.79 and 0.06, respectively). In the restricted cubic spline regression analysis, the positive dose–response association between waist-to-BMI ratio and MACE persisted across all the range of waist-to-BMI ratio. The positive dose–response association was non-linear with a much steeper increase in the risk of MACE for waist-to-BMI ratio > 3.6 cm‧m^2^/kg. In conclusion, waist-to-BMI ratio may function as a positive predictor for the risk of MACE in established ASCVD patients.

## Introduction

Obesity is an independent risk factor for cardiovascular diseases, such as hypertension, coronary artery disease (CAD), stroke and heart failure^1,2^. The clinical and epidemiological data indicate prevalence of modifiable risk factors for atherosclerotic cardiovascular disease (ASCVD), including hypertension, diabetes mellitus, and dyslipidemia, is higher in overweight and obese individuals^3–5^. While BMI is widely accepted and applied to evaluate obesity, a limitation of its use is that it cannot distinguish between fat mass, fat-free mass and lean mass^1^ and differentiate the distribution of subcutaneous or visceral fat. Emerging evidence has shown a phenomenon, called ‘obesity paradox’, in patients with established ASCVD^6–8^, in which the BMI-mortality curve is U-shaped. That is, overweight and mild to moderately obese patients have a better prognosis than patients with normal BMI and severe obesity.

It has been well known that increased visceral adipose tissue is associated with vascular inflammation, and subsequent atherosclerosis^9,10^. Visceral or central obesity plays an important role in ASCVD patients. Waist indices, such as waist circumference (WC) and waist-hip ratio (WHR), have been the two most common measures for central obesity. Both measures correlate with visceral adipose tissue and are associated with increased incident cardiovascular events^11,12^, and seem to be better anthropometric parameters for the prediction of cardiovascular outcome than BMI. However, WC alone is theoretically unable to allow comparison between individuals with different body size and mass. Hip circumference is technically more difficult to assess, and this made WHR a less reliable measure than WC^12^. Recently, two new WC-based anthropometric indices, body roundness index (BRI) and a body shape index (ABSI), have been proposed. BRI was developed by Thomas et al.^13^, and is calculated as 364.2–365.5x(1-((0.5xWC/π)^2^/(0.5xheight)^2^))^1/2^. One study has shown that BRI could identify cardiovascular disease and risk factors, but was not superior compared to traditional anthropometric indices like BMI and WC^14^. In 2012, Krakauer and Krakauer developed ABSI base on the allometric power law analysis to normalize WC for weight and height (and thus to BMI). It is defined as WC/(BMI^2/3^xheight^1/2^) and was designed to be independent from BMI^15^. ABSI has been demonstrated to predict premature mortality and cardiovascular risk in some studies^15–17^. However, both indices are too complicated to calculate and inconvenient for daily practice.

Therefore, we proposed a new and simplified waist-based index, waist-to-BMI ratio. In this new index, WC represents the visceral adiposity, and BMI is used for normalization of body size and mass. This study hypothesized the waist-to-BMI ratio as a good positive measure for central obesity, and a good anthropometric tool in patients with ASCVD. To test this hypothesis, the waist-to-BMI ratio was evaluated and compared with traditional anthropometric measures and newer waist-based indices in predicting the cardiovascular outcome in patients with established ASCVD from the Taiwanese Secondary Prevention for patients with AtheRosCLErotic disease (T-SPARCLE) Registry.

## Methods

### Study population

The study was conducted using the T-SPARCLE Registry, a multi-center prospective observational registry which involves 14 medical centers in Taiwan^18^. Briefly, the registry recruits and follows up a large population of patients with established ASCVD receiving secondary prevention therapy. Adult patients aged > 18 years with stable ASCVD, including CAD, cerebrovascular disease and peripheral artery disease, were enrolled. CAD was defined as the presence of coronary stenosis > 50% by coronary angiography, having a history of myocardial infarction, or angina with a positive stress test. Cerebrovascular disease was defined as cerebral infarction, or transient ischemic attack with carotid artery stenosis > 70% by ultrasound. Peripheral artery disease was defined as peripheral atherosclerosis with ischemic symptoms and confirmed by ankle-brachial index, Doppler ultrasound, or angiography. Patients were excluded if having a severe heart failure (New York Heart Association functional class III-IV), hemodynamically significant valvular or congenital heart disease, life expectancy of less than 6 months, neurocognitive or psychiatric condition, or being treated with immunosuppressive agents. Patients with acute stroke, acute myocardial infarction, or coronary revascularization within the last 3 months or being scheduled to receive coronary bypass graft surgery were also excluded. The study was approved by the Joint Institutional Review Board, Taiwan, R.O.C. for each participating hospital, and informed consent were obtained from all patients. All methods were performed in accordance with the relevant guidelines and regulations.

### Clinical and demographic data

After the enrollment, patients’ baseline characteristics, including anthropometric measures, vital signs, medical history and current medications, were recorded, and the lipid profiles, liver enzymes and creatine phosphokinase levels were evaluated. Patients were followed up at the 6th, and 12th month, and every year thereafter. At each follow-up, anthropometric measures, vital signs, medications, laboratory data, and major adverse cardiovascular event (MACE) were obtained. The baseline lipid profile, including total cholesterol (TC), high-density lipoprotein cholesterol (HDL-C), low-density lipoprotein cholesterol (LDL-C) and triglyceride (TG), were checked. The subjects’ fasting sugar, liver function and renal function were also measured at the same time.

### Study outcomes

The end point of this study was the composite MACE, defined as cardiovascular death, nonfatal myocardial infarction or stroke, or cardiac arrest with resuscitation.

### Statistical analysis

All continuous variables were expressed as mean ± standard deviation (SD) and dichotomous variables as percentage. Association between various anthropometric indices were examined using the Pearson correlation coefficients. Multivariable Cox proportional hazards regression models were used to estimate the hazard ratio (HR) and 95% confidence interval of categorical BMI for MACE with the normal weight group as the reference, adjusting for potential risk factors, such as age, sex, history of cigarette smoking, medical history of hypertension, diabetes mellitus, heart failure, CAD and stroke or transient ischemic attack, previous coronary intervention, statin use and its intensity, other medication (such as antiplatelet agent, angiotensin-converting enzyme inhibitor or angiotensin II receptor blocker, beta-blocker), level of non-HDL-C and estimated glomerular filtration rate . BMI was divided into categories according to the definition of WHO for Asian populations^19^: underweight (BMI < 18.5 kg/m^2^), normal weight (18.5 kg/m^2^ ≤ BMI < 23 kg/m^2^), overweight (23 kg/m^2^ ≤ BMI < 27.5 kg/m^2^), and obese (27.5 kg/m^2^ ≤ BMI). Dose–response association between risk of MACE and each continuous anthropometric measure, including weight, BMI, WC, WHR, BRI, ABSI and the waist-to-BMI ratio, was examined using the Cox proportional hazards regression model. We used restricted cubic spline regression to investigate the potential nonlinear relationship between each anthropometric measure and risk of MACE, and the likelihood ratio test was used to test for nonlinearity^20^. All statistical analyses were performed with the Statistical Analysis System (SAS) version 9.4 (SAS Institute, Cary, NC) and R version 3.5.2 (The R Foundation for Statistical Computing, 2018). A *p* value < 0.05 was considered significant.

### Missing data imputation

Approximately 5% of the enrolled patients had missing data on weight, BMI, and TG, and 8–12% in LDL-C, HDL-C, non-HDL-C, or estimated glomerular filtration rate. The missing rate of WC, and therefore WHR and waist-to-BMI ratio, was around 20%. Multiple imputation (by PROC MI procedure in SAS) was used to handle all the missing data. Under the assumption of missing at random, multiple imputation was considered as the most effective way of treating missing data that may provide unbiased results^21^. In this study, 20 plausible values were generated and imputed for each unobserved data point, resulting in 20 complete data sets for pooled analysis. Cox proportional hazards model was then estimated for each complete data set, and then used PROC MIANALYZE procedure in SAS to combine results from the twenty Cox models.

## Results

A total of 6,920 patients with ASCVD, enrolled from January 2010 to November 2014, were included in this analysis, with a mean age of 65.9 (SD 11.7) years, 73.9% males, and a mean BMI of 26.3 (SD 3.8) kg/m^2^ at baseline. Among them, 1.1% were underweight, 16.7% were normal weight, 48.5% were overweight, and 33.7% were obese. The mean weight, WC and WHR were 69.8 (SD 12.4) kg, 92.6 (10.1) cm and 0.93 (0.07) respectively. Of the 6,920 patients, 6,235 (90.1%) had CAD, most were acute coronary syndrome (77.2%), and 4862 (70.3%) received lipid-lowering therapy. Other baseline clinical and demographic characteristics were listed in Table 1. At follow-up, 58.2%, 60.6%, and 67.5% of the ASCVD patients achieved the optimal serum level of LDL-C < 100 mg/dL, non-HDL-C < 130 mg/dL, and TG < 150 mg/dL (the target of the guideline of national health insurance of Taiwan, during the study period), respectively. Correlation coefficients of each anthropometric index with BMI in this study cohort are shown in Table 2.

**Table 1:** Baseline characteristics of patient study.

Patient characteristics, n (%)	Patients
	n = 6920
Age, mean ± SD	65.9 ± 11.7
**Sex**	
Male	5111 (73.9)
Female	1809 (26.1)
Weight (kg), mean ± SD	69.8 ± 12.4
Height (cm), mean ± SD	162.8 ± 8.2
**BMI (kg/m**^**2**^**), mean ± SD**	26.3 ± 3.8
< 18.5	74/6589 (1.1)*
18.5–23	1100/6589 (16.7)*
23–27.5	3193/6589 (48.5)*
≥ 27.5	2222/6589 (33.7)*
Waist circumference (cm), mean ± SD	92.6 ± 10.1
Hip circumference (cm), mean ± SD	99.3 ± 8.5
Waist-hip ratio, mean ± SD	0.93 ± 0.07
Body roundness index, mean ± SD	4.83 ± 1.36
A body shape index (m^11/6^/kg^2/3^), mean ± SD	0.082 ± 0.006
Waist-to-BMI ratio (cm·m^2^/kg), mean ± SD	3.55 ± 0.35
Systolic BP (mmHg), mean ± SD	132.6 ± 18.1
Diastolic BP (mmHg), mean ± SD	76.0 ± 11.4
Cigarette smoking	3062/6910 (44.3)*
Hypertension	4978/6914 (72.0)*
Diabetes mellitus	3180/6468 (49.2)*
Total cholesterol (mg/dL), mean ± SD	170.5 ± 38.7
Triglyceride (mg/dL), mean ± SD	140.3 ± 93.9
LDL-C (mg/dL), mean ± SD	97.9 ± 34.0
HDL-C (mg/dL), mean ± SD	44.9 ± 13.1
Non-HDL-C (mg/dL), mean ± SD	124.9 ± 37.3
**Coronary artery disease**	6235 (90.1)
Acute coronary syndrome	5341 (77.2)
Stable angina	894 (12.9)
**Cerebrovascular events**	1033 (14.9)
Ischemic stroke	823 (11.9)
Transient ischemic attack	210 (3.0)
Peripheral arterial disease	141 (2.0)
Previous coronary intervention	3587 (51.8)
Heart failure	755/6909 (10.9)*
**Chronic kidney disease (eGFR in mL/min)**	76.5 ± 26.9
Stage 1–2 (> 60)	4522/6198 (73.0)*
Stage 3 (31–60)	1490/6198 (24.0)*
Stage 4–5 (≤ 30)	186/6198 (3.0)*
**Lipid-lowering therapy**	4862 (70.3)
With statin	4587 (66.3)
Low-intensity	714 (10.3)
Moderate-intensity	3631 (52.5)
High-intensity	242 (3.5)
With fibrate	399 (5.8)
Anti-platelet agents	5852 (84.6)
ARB or ACE inhibitors	3974 (57.4)
Beta blockers	3761 (54.3)

Data are presented as number (percentage) or mean ± SD.

*ACE* Angiotensin-converting enzyme, *ARB* Angiotensin II receptor blocker, *BMI* Body mass index, *BP* Blood pressure, *eGFR* Estimated glomerular filtration rate, *HDL-C* High-density lipoprotein cholesterol, *LDL-C* Low-density lipoprotein cholesterol.

*Means that there were missing values, and the actual numerators and denominators are presented.

**Table 2:** Correlations between BMI and various anthropometric indices.

	BMI	Weight	WC	WHR	BRI	ABSI	Waist-to-BMI ratio
BMI	1	0.816	0.721	0.305	0.727	− 0.233	− 0.640
Weight	0.871	1	0.736	0.337	0.462	− 0.179	− 0.374
WC	0.754	0.757	1	0.590	0.879	0.455	0.049
WHR	0.353	0.311	0.585	1	0.511	0.443	0.217
BRI	0.772	0.606	0.936	0.571	1	0.442	− 0.079
ABSI	− 0.141	− 0.105	0.497	0.451	0.470	1	0.848
Waist-to-BMI ratio	− 0.621	− 0.436	0.012	0.148	− 0.084	0.825	1

Right side (above diagonal) shows correlations of the raw values; left side (below diagonal) shows correlations of the z scores relative to age- and sex-specific means.

*ABSI* A body shape index, *BMI* Body mass index, *BRI* Body roundness index, *WC* Waist circumference, *WHR* Waist-hip ratio.

The 6,920 ASCVD patients were followed up for a median of 2.5 years, and 227 patients experienced MACE. The incident rate of MACE in our ASCVD patients was 1.25 (95% CI, 1.10–1.42) per 100 person-years. In the multivariable Cox regression analysis for categorical BMI, overweight ASCVD patients had the lowest hazard of MACE (adjusted HR 0.61, 95% CI 0.43–0.85, *p* = 0.004) (Table 3).

**Table 3:** Multiple Cox proportional hazards regression model for categorical BMI to predict the risk of MACE in ASCVD patients.

	Model with categorical BMI (Sample size after imputation n = 6920)		
	β	Hazard ratio (95% CI)	*p* value
Age	0.02	1.02 (1.00–1.03)	0.02
Male (vs. female)	− 0.02	0.98 (0.69–1.39)	0.92
**BMI (vs 18.5 ≤ BMI < 23)**			
BMI < 18.5	0.46	1.58 (0.71–3.54)	0.26
23 ≤ BMI < 27.5	− 0.50	0.61 (0.43–0.85)	0.004
BMI ≥ 27.5	− 0.34	0.71 (0.49–1.03)	0.07
History of hypertension	0.01	1.01 (0.74–1.38)	0.96
History of diabetes mellitus	0.38	1.46 (1.11–1.93)	0.01
History of heart failure	0.69	1.99 (1.43–2.76)	< 0.0001
History of coronary artery disease	0.38	1.46 (0.84–2.53)	0.18
Previous coronary intervention	0.34	1.40 (1.03–1.90)	0.03
Ischemic stroke or TIA	0.40	1.48 (0.98–2.24)	0.06
History of cigarette smoking	0.28	1.32 (0.97–1.80)	0.07
**Statin intensity (vs. Moderate-intensity)**			
No statin use	0.22	1.24 (0.92–1.68)	0.16
Low-intensity	− 0.19	0.83 (0.51–1.35)	0.45
High-intensity	− 0.95	0.39 (0.12 − 1.22)	0.11
Fibrate	− 0.45	0.64 (0.32–1.26)	0.20
Anti-platelets therapy	0.07	1.07 (0.73–1.58)	0.71
ARB or ACE inhibitor	0.04	1.04 (0.79–1.38)	0.76
Beta-blocker	− 0.36	0.70 (0.53–0.92)	0.01
**eGFR (vs. eGFR > 60 ml/min)**			
30 < eGFR ≤ 60 ml/min	0.52	1.69 (1.23–2.31)	0.001
eGFR ≤ 30 ml/min	1.36	3.91 (2.42–6.34)	< 0.0001
**Non-HDL-c level (vs. < 100 mg/dL)**			
100 ≤ Non-HDL-c < 130	0.25	1.28 (0.87–1.90)	0.21
130 ≤ Non-HDL-c < 160	0.43	1.54 (1.02–2.32)	0.04
Non-HDL-c ≥ 160	0.47	1.60(1.02–2.51)	0.04

*ACE* Angiotensin-converting enzyme, *ARB* Angiotensin II receptor blocker, *BMI* Body mass index, *eGFR* Estimated glomerular filtration rate, *HDL-C* High-density lipoprotein cholesterol, *TIA* Transient ischemic attack.

**Figure 1:**
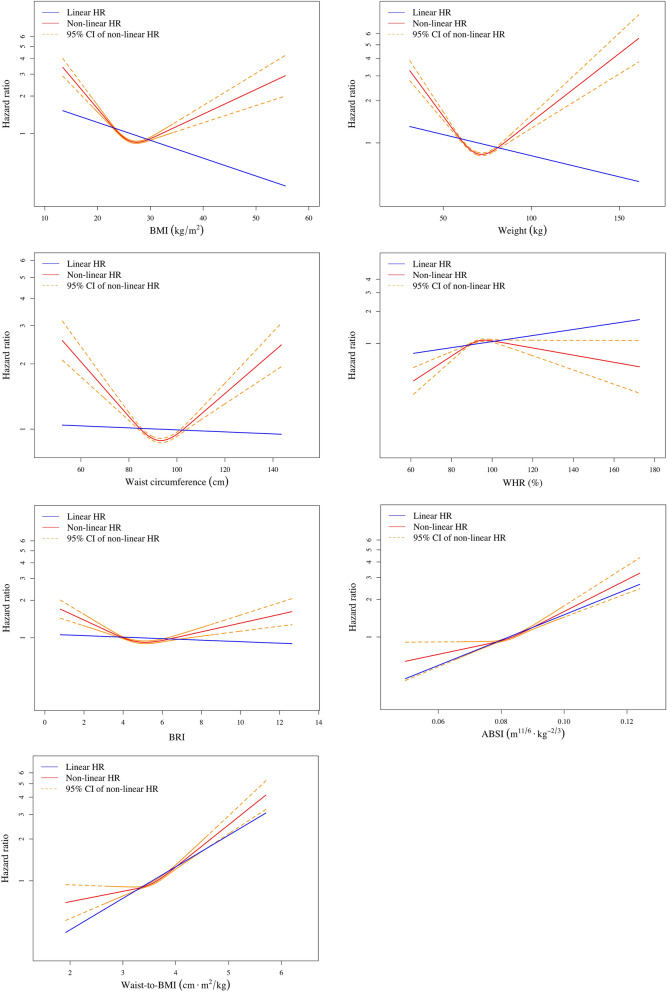
Dose–response curves for various anthropometric parameters and risk of MACE. The red line and orange dashed line represent the point estimates and 95% confidence intervals from the non-linear analysis using the cubic spline regression. The blue line represents the point estimates from the linear analysis using the Cox proportional hazards regression.

When analyzing continuous anthropometric parameters, multivariable Cox proportional hazards regression showed a significant positive association between waist-to-BMI ratio and the risk of MACE after adjusting for the covariates (adjusted HR 1.69 per cm·m^2^/kg increase in waist-to-BMI ratio, 95% CI 1.12–2.49, *p* = 0.01). Although non-significant, the hazard of MACE increased positively with ABSI with a trend (*p* = 0.06). Traditional anthropometric parameters, such as BMI, weight, WC and WHR, or the newer waist-based index, BRI, however, did not show significant associations with the risk of MACE (*p* = 0.09, 0.30, 0.89, 0.54, and 0.79, respectively) (Table 4). In the restricted cubic spline regression analysis, U-shaped associations were observed between traditional anthropometric parameters or BRI and the risk of MACE (Fig. 1). While dose–response association was also nonlinear between ABSI or waist-to-BMI ratio and the risk of MACE (the test for nonlinearity was statistically significant, *p* = 0.049 and < 0.001, respectively), positive associations persisted across all range of ABSI and waist-to-BMI ratio. By estimating the point of inflection from the figure of the restricted cubic spline regression analysis, a much steeper increase in the risk of MACE was found for waist-to-BMI ratio > 3.6 cm·m^2^/kg (Fig. 1).

**Table 4:** Dose response association between various anthropometric parameters and risk of MACE in ASCVD patients examined using the Cox proportional hazards regression model.

	Sample size after imputation n = 6920			
	β	Adjusted hazard ratio	95% CI	*p* value
BMI	− 0.03	0.97	0.93–1.01	0.09
Weight	− 0.01	0.99	0.98–1.01	0.30
WC	− 0.001	1.00	0.98–1.01	0.89
WHR	0.01	1.01	0.99–1.03	0.54
BRI	− 0.01	0.99	0.89–1.09	0.79
ABSI	0.23	1.26	0.99–1.62	0.06
Waist-to-BMI ratio	0.52	1.69	1.13–2.52	0.01

Each model was adjusted for age, sex, history of cigarette smoking, medical history of hypertension, diabetes mellitus, heart failure, CAD and stroke or transient ischemic attack, previous coronary intervention, statin use and its intensity, other medication, levels of non-HDL cholesterol and levels of eGFR.

*ABSI* A body shape index, *BMI* Body mass index, *BRI* Body roundness index, *WC* Waist circumference, *WHR* Waist-hip ratio.

## Discussion

Obesity paradox was observed again in this study, and the results were consistent with previous studies when analyzing categorical BMI. Overweight ASCVD patients had the lowest risk of MACE during the follow-up period. Both the Cox proportional hazards model and cubic spline regression analysis showed a positive dose–response association between waist-to-BMI ratio and the risk of MACE, although the association was nonlinear. The shape of positive association between ABSI and the risk of MACE was similar with waist-to-BMI ratio by the cubic spline regression analysis although the positive association was non-significant by the Cox proportional hazards model. BMI and other traditional anthropometric measures, such as weight, WC and WHR, or the newer waist-based index, BRI, showed U-shaped associations with the cardiovascular outcome. We found that waist-to-BMI ratio is a good positive anthropometric measure to predict cardiovascular outcomes in patients with ASCVD, especially when the ratio is above 3.6 cm·m^2^/kg, which can be an easy, convenient, and useful tool for the clinical practice.

Although the World Health Organization defines obesity as abnormal or excessive fat accumulation that may impair health and suggest that BMI is only a rough guide to diagnose overweight and obesity, it is still widely used in epidemiological surveys and clinical practice due to its simplicity^22,23^. Association between BMI and first cardiovascular event and mortality in the general population have been confirmed by a large number of studies^24–27^. Therefore, BMI is widely used to predict cardiovascular outcomes in primary prevention at the population level. However, the ‘obesity paradox’ phenomenon of BMI^6–8^ in patients with established ASCVD could limit its application as an anthropometric tool for predicting cardiovascular outcomes. With the use of BMI in the present study, overweight ASCVD patients had the lowest risk for MACE, and the revealed U-shaped association is consistent with that of previous studies. The explanation of the obesity paradox may be the inherent limitations of clinical studies and the BMI as an obesity index^28^. The obesity paradox is observed by epidemiological studies, which could be biased by reverse causality, selection bias, inadequate follow-up periods, etc. BMI cannot distinguish between fat and lean mass, and it does not account for fat distribution, such as visceral fat or peripheral fat^29–31^. Individuals with similar BMI may have a different body fat percentage and fat distribution. Romeo-Corral et al. has demonstrated the poor diagnostic performance of BMI to discriminate between body fat and lean mass and to detect obesity in patients with CAD^32^. BMI is a crude measurement of total adiposity and does not seem to be a good clinical anthropometric tool for predicting CV outcome in ASCVD patients. A similar U-shaped association of BMI with prognosis has been also observed in patients with some other diseases, such as hypertension, heart failure, atrial fibrillation and chronic pulmonary disease although the reason might be not the same with that for ASCVD^1^.

Increased visceral adipose tissue is associated with the incidence of traditional cardiovascular risk factors, such as hypertension, hyperlipidemia, diabetes mellitus, and metabolic syndrome^9,33,34^. Besides, visceral adipose tissue plays an important role in the vascular inflammation. Image and autopsy studies demonstrated an association of abdominal visceral fat with coronary artery atherosclerosis^35,36^, the prevalent cardiovascular disease^37,38^, the severity of coronary artery disease^39^ and MACE^40^. Since regional adiposity is more important in ASCVD patients, a pertinent anthropometric measure is necessary for clinical use.

WC and WHR are thought to be better anthropometric measures than BMI to evaluate body fat distribution^41^ and can be surrogates for central or visceral fat adipose tissue^42^. Mounting evidence suggests that WC and WHR are associated with the risk of cardiovascular events and may be a better anthropometric measure to predict those events^12,43,44^. However, there is a lack of robust data about the relationship between central obesity and cardiovascular outcomes in patients with established ASCVD. One study demonstrated that central obesity was associated with adverse prognosis in patients with CAD, and highlighted the importance of central obesity in mortality risk assessment in this group of patients^41^. Our study showed that WC failed to predict cardiovascular outcome in patients with ASCVD. WC alone is theoretically unable to allow comparison between individuals with different body size and mass. Although the past studies showed that WC correlated well with BMI, a considerable variation in WC was noted for any given BMI value^45,46^. This means that individuals with the same BMI may have different WC, the different level of central obesity. One study has showed that patients with normal BMI but central obesity possess the worst cardiovascular outcome^47^. On the other hand, individuals with the same WC may have different BMI. Specifically, someone with lower BMI might have less lean mass but higher abdominal fat. Hence, individuals with the same WC might have different level of abdominal fat distribution. This implicated that WC should be interpreted in the context of BMI.

In this study, waist-to-BMI ratio was proposed to be a single and simple clinical anthropometric measure and adiposity index. Waist-to-BMI ratio incorporates the cardiovascular risk effect of regional adiposity WC with the application of BMI for normalization of body size and mass, which would be a better adiposity index for fat distribution or central obesity to predict cardiovascular outcomes in ASCVD patients. After normalizing WC by BMI, waist-to-BMI ratio was shown to have a significantly and consistently positive association with cardiovascular outcomes. This study also showed that the hazard of MACE increased positively with all range of ABSI, a recently proposed waist-based index, in the cubic spline regression analysis. With similar concept with waist-to-BMI ratio, ABSI was proposed to take body size and mass into account, and was designed to be independent of BMI base on the allometric power law analysis^15^. It has been shown to predict cardiovascular risk in some studies^48–50^. However, ABSI is too complicated to calculate for daily practice. The results of this study showed that waist-to-BMI ratio was not inferior, or even better, than ABSI in prediction of cardiovascular outcomes. This study demonstrated the waist-to-BMI ratio could be a good and simple anthropometric tool for predicting cardiovascular outcomes in established ASCVD patients, especially when the ratio is higher than 3.6 cm‧m^2^/kg.

There are some limitations in this study that should be considered. First, this study did not evaluate the effect of cardiorespiratory fitness. Increasing evidence showed cardiorespiratory fitness is a strong prognostic factor in CVD, which may interact with the prediction power of anthropometric measures^30,51^. Second, the follow-up time was not long. Third, the sample size of this study was moderate, and the study sample was limited to Asian population. Further study with a larger sample size, and extended to other ethnic groups are required to address this issue.

In conclusion, this is the first study to examine waist-to-BMI ratio as an anthropometric measure to predict CV outcome. We found waist-to-BMI ratio to be a better positive predictor for MACE in established ASCVD patients than traditional anthropometric parameters. Our finding provided an easy, convenient, and useful anthropometric tool in patients with ASCVD in daily clinical practice.
